# Protected insect species in Italy: occurrence data from a 10-year citizen science initiative

**DOI:** 10.3897/BDJ.13.e151742

**Published:** 2025-05-07

**Authors:** Alice Lenzi, Silvia Gisondi, Marco Bardiani, Sönke Hardersen, Emanuela Maurizi, Fabio Mosconi, Gianluca Nardi, Alessandro Campanaro

**Affiliations:** 1 Council for Agricultural Research and Economics, Research Centre for Plant Protection and Certification, Florence, Italy Council for Agricultural Research and Economics, Research Centre for Plant Protection and Certification Florence Italy; 2 University of Siena, Department of Life Sciences, Siena, Italy University of Siena, Department of Life Sciences Siena Italy; 3 Council for Agricultural Research and Economics, Research Centre for Plant Protection and Certification, Rome, Italy Council for Agricultural Research and Economics, Research Centre for Plant Protection and Certification Rome Italy; 4 Centro Nazionale Carabinieri Biodiversità “Bosco Fontana”, Marmirolo, Italy Centro Nazionale Carabinieri Biodiversità “Bosco Fontana” Marmirolo Italy

**Keywords:** Coleoptera, conservation, dataset, entomology, GBIF repository, Habitats Directive, Lepidoptera, Odonata, Orthoptera, volunteering

## Abstract

**Background:**

Occurrence data provide an important baseline for the planning of conservation strategies and for the protection of species and habitats. However, collecting such data usually requires energy and it is time-consuming. Recently, citizen science has been shown to be a suitable approach for the study and monitoring of biodiversity, as it allows for the collection of a large number of records, distributed spatially and over time. Additionally, this approach enable the generation of new knowledge and fosters environmental awareness in the participating volunteers.

**New information:**

The present paper describes the data collected during the first citizenscience project on protected insect species in Italy. The dataset contains occurrence records of 31 taxa observed all over Italian national territory in 10 years for a total of 5,975 records. The aim of the project was to increase the knowledge, to document the distribution of the target taxa and to provide valuable data useful for the reporting of these insects as required by Articles 11 and 17 of the Habitats Directive.

## Introduction

In a changing world, exacerbated by the climate crises, environmental degradation and biodiversity loss (Pereira et al. 2012, Pereira et al. 2024), species distribution data are a valuable source of information that improves our knowledge on population dynamics (Samy et al. 2013, Isaac and Pocock 2015) and can provide a reliable baseline for conservation planning (Powney and Isaac 2015). Concerning insects, their taxonomic diversity, species distribution, local conservation status and population trends are still poorly known (Mora et al. 2011, Leandro et al. 2017, Wilson 2017), despite proofs of their dramatic decline in diversity and abundance (Hallmann et al. 2017, Hallmann et al. 2021, Wagner et al. 2021, Outhwaite et al. 2022). This does not only apply to hyper-diverse and little-studied groups, but also to more charismatic and easily detectable taxa, including those protected at national or international level and/or flagship/umbrella species. Importantly, these taxa are often employed as proxies for a rich community and as indicators for habitat conservation status (Caro 2011, Lindenmayer and Westgate 2020) and they are often targeted by conservation programmes and initiatives. In addition, the European Commission requires Member States to monitor biodiversity through constant surveillance (Art. 11 of Habitats Directive 92/43/ECC) of protected species and habitats (i.e. listed in Annexes I, II and IV of the Habitats Directive 92/43/ECC) and to report the results every 6 years (ex Art. 17 of Habitats Directive 92/43/ECC). In order to comply with these obligations, a large number of spatially and temporally distributed records are needed, which requires energy- and time-consuming efforts for data collection and analysis.

Since the late twentieth century, an engaging and promising tool for collecting a large number of species records is represented by the citizen science (CS) approach. This consists in involving volunteers from the general public in scientific processes under the coordination of experts (ECSA 2015, Eitzel et al. 2017, Shanley et al. 2019). Such initiatives find applications in many scientific fields (Bonney et al. 2009, Kullenberg and Kasperowski 2016, Chandler et al. 2017, Pocock et al. 2018), with a remarkable number of emerging projects (most are accessible through European or global repositories, such as eu-citizen.science and SciStarter). A small number of these CS projects are dedicated to the study of insects, often focusing on charismatic groups (Sheard et al. 2024) which are easily detectable and identifiable also by non-experts after specific training. Amongst CS projects that focus on insects, most target pollinators (e.g. bees, bumblebees and butterflies) (van Swaay et al. 2008, Dennis et al. 2017, Schultz et al. 2017, Barahona-Segovia et al. 2022) and some large-sized and conspicuous Coleoptera (Percy et al. 2000, Losey et al. 2012, Mason et al. 2015, Campanaro et al. 2017, Thomaes et al. 2021, Bardiani et al. 2022, Lenzi et al. 2023). Considering these groups, many studies have shown that the involvement of volunteers in collecting occurrence data of observed individuals provides important information on the distribution of species as well as their habitat requirements (Campanaro et al. 2017, Tiago et al. 2017, Soroye et al. 2018, Brown and Williams 2018, Matutini et al. 2021, Lenzi et al. 2022, Redolfi De Zan et al. 2023). Accordingly, CS has been recognised as a valid approach, complementary to traditional science, since it allows gathering data faster and on a wider scale, thanks to the involvement of a large number of participants (Gardiner et al. 2012, Dennis et al. 2017, Soroye et al. 2018, Robinson et al. 2020, Fontaine et al. 2021).

An important issue concerning CS projects is the reliability of the recorded data, commonly ensured by a data quality assessment process by expert scientists (Kosmala et al. 2016, Aceves‐Bueno et al. 2017). For instance, in some projects the verification of species identification is carried out by the community of volunteers or by advanced technologies (e.g. artificial intelligence) (Leibovici et al. 2017, Aristeidou et al. 2021, López-Guillén et al. 2024), while other projects employ a team of experts to ensure that the collected data are correct (Wiggins et al. 2011, Tulloch et al. 2013, Campanaro et al. 2017, Flaminio et al. 2021). Once data are collected, it is recommended to make them publicly available, together with the metadata, if possible and results should be published in an open access format (Ten Principles of Citizen Science, ECSA (2015), Cooper et al. (2021)). For this reason, it is increasingly common and recomended to share data and results from CS projects, possibly following the FAIR principles (i.e. Findable, Accessible, Interoperable and Reusable, Wilkinson et al. (2016), Boeckhout et al. (2018)), while complying with any possibile restrictions, for example, sensitive data on rare and protected species, privacy data.

This paper presents and describes the dataset from a citizen initiative called MIPP/InNat, developed in the framework of two projects (LIFE MIPP "Monitoring of Insects with Public Participation" and InNat "Promozione della Rete Natura 2000 e il Monitoraggio a scala nazionale di specie di insetti protetti"), which is accessible through GBIF (Global Biodiversity Information Facility). The paper also provides some descriptive statistics. The CS-based data collection, which started in 2014 and ended in 2024, was supported by funding from different international and national sources and focused on the collection of occurrence data of selected and protected insect taxa.

## General description

### Purpose

The purpose of this publication is to share and make freely available occurrence data on protected insect species (i.e. listed in Annexes II and IV of the Habitats Directive) recorded in Italy and collected during the above-mentioned 10-year CS initiative. We describe the dataset consisting of occurrence data of 31 selected species belonging to four insect orders: Coleoptera (6 taxa), Lepidoptera (16 taxa), Odonata (7 taxa) and Orthoptera (2 taxa).

These records contribute to further the knowledge on the distribution of the target insects, thus providing an additional tool for planning specific conservation actions (Wilkinson et al. 2016, Boeckhout et al. 2018). Indeed, these data are also included and stored in the database of the Italian National Network of Biodiversity (NNB) which is one of the main sources that are considered for the reporting required by Arts. 11 and 17 of the Habitats Directive.

## Project description

### Title

MIPP/InNat initiative.

### Study area description

Data were collected in all of Italy.

### Design description

The MIPP/InNat initiative engaged volunteers in collecting occurrence data of protected insects.

### Funding

The MIPP/InNat initiative was funded under different projects:

- the LIFE project MIPP (LIFE11 NAT/IT/000252), “Monitoring of insects with public participation,” co-funded by the European Commission [2012–2017];
- the national agreement entitled InNat "Promozione della Rete Natura 2000 e il Monitoraggio a scala nazionale di specie di insetti protetti" funded by the former Direzione Generale per la Protezione della Natura e del Mare – Ministero dell'Ambiente e della Tutela del Territorio e del Mare and the Comando Unità Forestali, Ambientali e Agroalimentari Carabinieri – Comando Carabinieri per la Tutela Della Biodiversità e dei Parchi [2017–2018];
- the national agreement entitled START2000 "Sviluppo di strumenti di coordinamento finalizzati all'attuazione degli obiettivi e delle misure di conservazione nei siti Natura 2000 compresi nelle Riserve ed altre Aree demaniali gestiti dall'Arma dei Carabinieri" funded by the former Direzione Generale per la Protezione della Natura e del Mare – Ministero dell'Ambiente e della Tutela del Territorio e del Mare and the Comando Unità Forestali, Ambientali e Agroalimentari Carabinieri – Comando Carabinieri per la Tutela Della Biodiversità e dei Parchi [2019–2022];
- the National Recovery and Resilience Plan (NRRP), Mission 4 Component 2 Investment 1.4 – Call for tender № 3138 of 16 December 2021, rectified by Decree № 3175 of 18 December 2021 of Italian Ministry of University and Research funded by the European Union – NextGenerationEU; Award Number: Project code CN_00000033, Concession Decree № 1034 of 17 June 2022 adopted by the Italian Ministry of University and Research, CUP B83D21014060006, Project title “National Biodiversity Future Center – NBFC” [2022–2024].

## Sampling methods

### Sampling description

Volunteers were asked to use the project websites of the two projects or a specific app for Android and iOS (called MIPP and then InNat, discontinued) to upload pictures of observed target insects. The volunteers were free as to where or when to perform the observations. The following information was required during the uploading process: 1) tentative identification (species or genus level); 2) date and hour of the sighting; 3) geografic coordinates (WGS84, decimal); 4) location; 5) additional notes. The geographic coordinates were collected using the GPS sensor of the smartphone, manually by using Google Maps (which was accessible through the platforms) or by entering coordinates by hand. These precise coordinates were thus stored in the InNat database and were downloadable upon request during the project. Finally, the volunteers provided their e-mail addresses and a nicknames (no sensitive data were collected), in order to receive any feedback about their record.

### Quality control

In order to aid the volunteers in recognising and finding the various insects, fact sheets were provided, which included information on the morphology and ecology of the target insects.

The platform was developed using MySQL and was accessible with credentials by the project staff. Once a record was uploaded, it entered the project database with the initial status "pending". Subsequently, the experts validated the associated images and the information provided. If the record was "confirmed", it became visible on the project website to everyone. When no images were provided, the volunteers were contacted by the project team and were asked to provide further details on the record. Data without images were classified as valid only after careful consideration. This strict approach resulted in only approximately 3% of records without images being confirmed. Only validated and confirmed records were analysed and published as a dataset in the GBIF repository and are here described. Moreover, even if the volunteers had not been asked to provide the number of observed individuals during data submission, in the present dataset, an additional column about the number of the individuals observed for each record is presented and this was obtained by the project staff by counting the number of specimens portrayed in each image recorded.

## Geographic coverage

### Description

**Figure 1.**
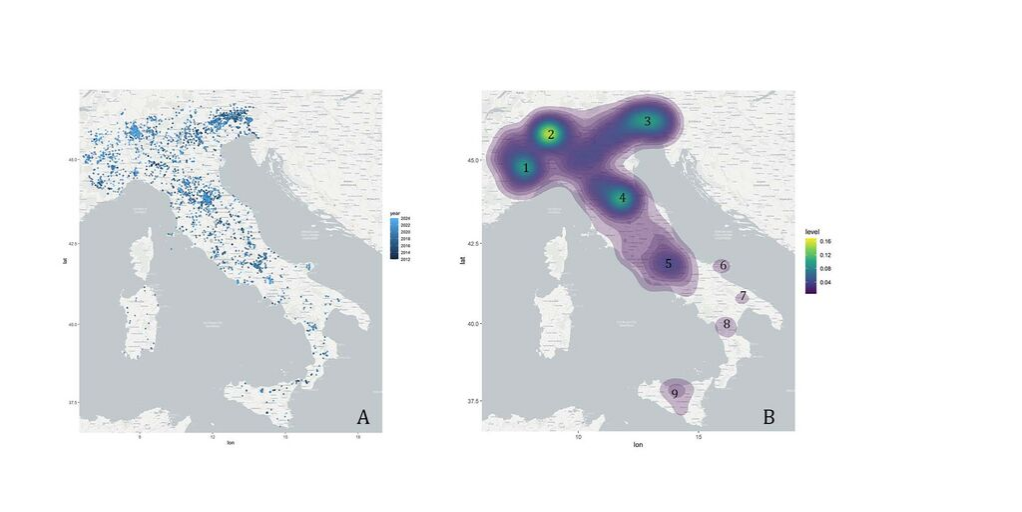
Geographic coverage of records. **A** The colour scale of the data points indicates the year of the sightings; a lighter colour indicates a more recent sighting, while a darker colour denotes older records; **B** Heatmap representing the concentration of records in Italy from the the lowest (in purple) to the highest (in yellow). The regions with the majority of data correspond to protected areas: Parchi Regionali dell'area Torinese (1), Parco Nazionale della Val Grande (2), Parco Regionale delle Prealpi Giulie e Parco Nazionale delle Dolomiti Bellunesi (3), Parco Nazionale delle Foreste Casentinesi (4), Parco Nazionale d'Abruzzo, Lazio e Molise (5), Parco Nazionale del Gargano (6), Parco Nazionale dell'Alta Murgia (7), Parco Nazionale del Pollino (8), Parco Regionale delle Madonie (9).

**Figure 2.**
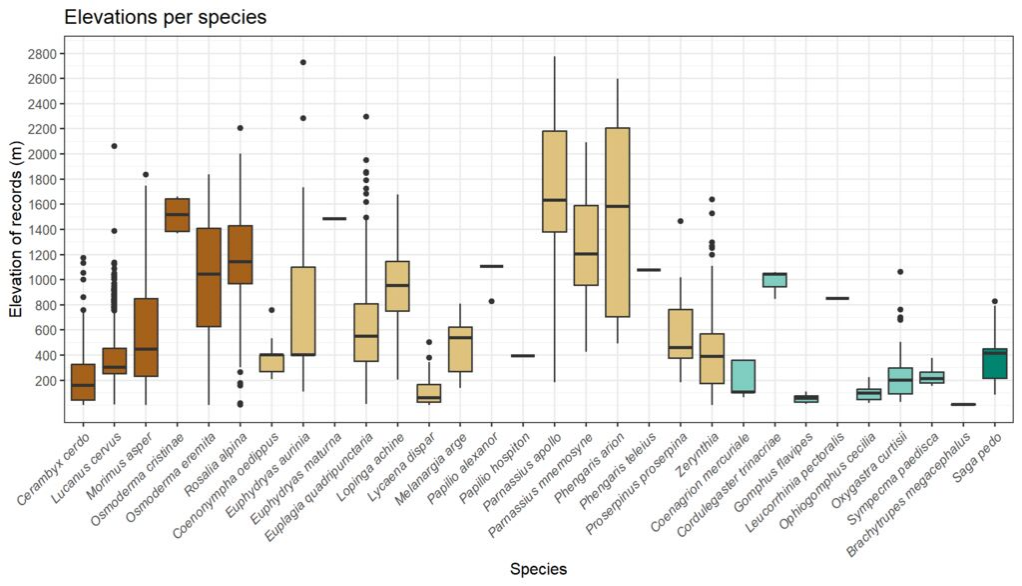
Boxplot showing the span of elevations at which target taxa were recorded.

The dataset contains records from all the Italian territory (Fig. 1). The majority of these records was collected in Central-Northern Italy possibly due to the fact that, amongst the ten most recorded species, three (i.e. *Lucanuscervus*, *Lopingaachine* and *Lycaenadispar*) are distributed exclusively in north-central regions. Moreover, five "hotspots" (Fig. 1, B) can be observed, corresponding to protected and mountain areas in which the target species are present with quite abundant populations. The altitudes at which the target insects were recorded (Fig. 2) are consistent with the species ecology. The lowest altitude recorded was 0 m a.s.l. (*Cerambyxcerdo*) and the highest altitude was 2,958 m a.s.l. (*Parnassiusapollo*).

### Coordinates

36.71 and 46.94 Latitude; 6.664 and 17.230 Longitude.

## Taxonomic coverage

### Description

At the beginning, LIFE MIPP covered nine target species. Subsequently, during the projects InNat and START2000, additional taxa were included, reaching gradually a total of 31 protected species listed in the Annexes II and IV of the Habitats Directive, belonging to Coleoptera, Lepidoptera, Odonata and Orthoptera (Table 1). Such later additions concern also species that are rather rare, with limited and scattered distribution and whose detection requires a somewhat higher level of volunteer training and experience (e.g. *Brachytrupesmegacephalus* (Lefèvre, 1827), *Papilioalexanor* Esper, 1800 and *Phengaristeleius* (Bergsträsser, 1779)). Despite the fact that fewer records were expected, these species were nevertheless included in the initiative as even the addition of a few occurrence data would be valuable for increasing the knowledge of their distribution. In general, the names of target species were based on those adopted by the Habitats Directive.

## Temporal coverage

**Data range:** 1973-7-13 – 2024-2-18.

### Notes

**Figure 3.**
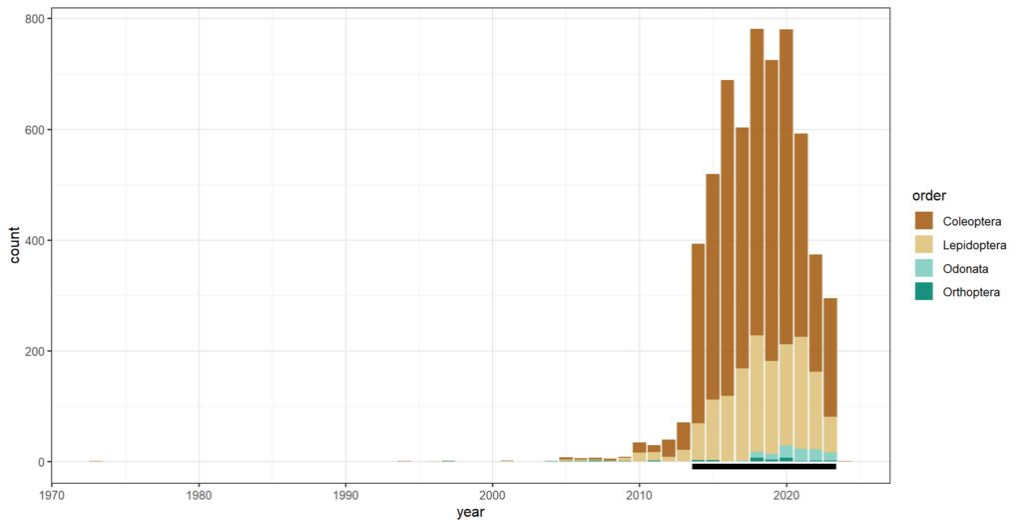
Temporal distribution of the occurrence records. The period during which the project was active is highlighted with a black bar. Orders are identified by different colours: Coleoptera in brown, Lepidoptera in light brown, Odonata in turquoise and Orthoptera in teal.

Although the data collection carried out by the MIPP/InNat initiative started in 2014, the dataset contains records from 1973 to 2024 (Fig. 3), because volunteers also provided occurrence data recorded previously. These data are considered useful and interesting for the purposes of the project and have been retained and published in the GBIF repository.

## Usage licence

### Usage licence

Other

### IP rights notes

Creative Commons Attribution-Non-Commercial 4.0 International Licence (CC-BY-NC 4.0)

## Data resources

### Data package title

Occurrences of protected species of insects in Italy

### Resource link

https://doi.org/10.15468/m5sfc6

### Number of data sets

1

### Data set 1.

#### Data set name

Occurrences of protected species of insects in Italy.

#### Download URL

https://cloud.gbif.org/eca/archive.do?r=protected_insects_of_italy

#### Description

The dataset “Occurrences of protected species of insects in Italy” was published on the repository Global Biodiversity Information Facility – GBIF under the Creative Commons Attribution-Non-Commercial 4.0 International Licence (CC BY-NC 4.0) as an open access file (Campanaro et al. 2024).

The file consists of occurrence data for 31 species protected under the Habitats Directive (92/43/ECC). Fields in the dataset follow the Darwin Core standard (Darwin Core Maintenance Group 2021, Wieczorek et al. (2012)).

**Figure 4.**
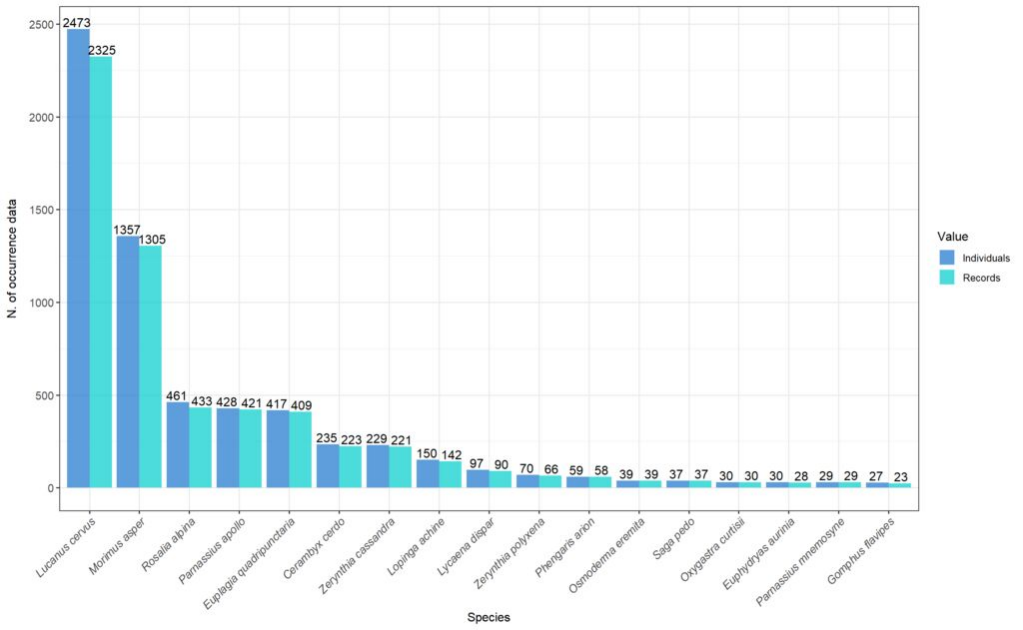
Barplot representing the total number of individuals and records for each insect taxon collected by the project. Only taxa with more than 20 records are plotted here.

The dataset contains 5,968 occurrence records for a total of 6,292 specimens (numbers of individuals were counted, based on the images sent by the volunteers) (Fig. 4): 4,577 Coleoptera, 1,568 Lepidoptera, 108 Odonata and 39 Orthoptera. Even if commonly lepidopterans are the most recorded taxa in CS projects, our result, highlighting a majority of coleopteran records, was quite expected. In fact, the LIFE MIPP was originally mostly focused on six saproxylic beetle species that had benefitted from further dissemination and scientific activity unlike the four butterflies and the one bush cricket. In addition to this, butterflies listed in the Habitats Directive Annexes are often rarer and with a more limited and/or scattered distribution in respect to coleopterans.

Approximately 97% of the records are associated with an image portraying the reported insect.

Data were collected by a total of 1,180 volunteers between 2014 and 2024 during different citizen-science projects, but, as specified in the "Temporal Coverage" section, some of the reported observations were collected before the start of the project (Fig. 3). The highest number of records was collected in the years 2018, 2019 and 2020, with a total of 781, 725 and 780 records, respectively (Fig. 3).

**Figure 5.**
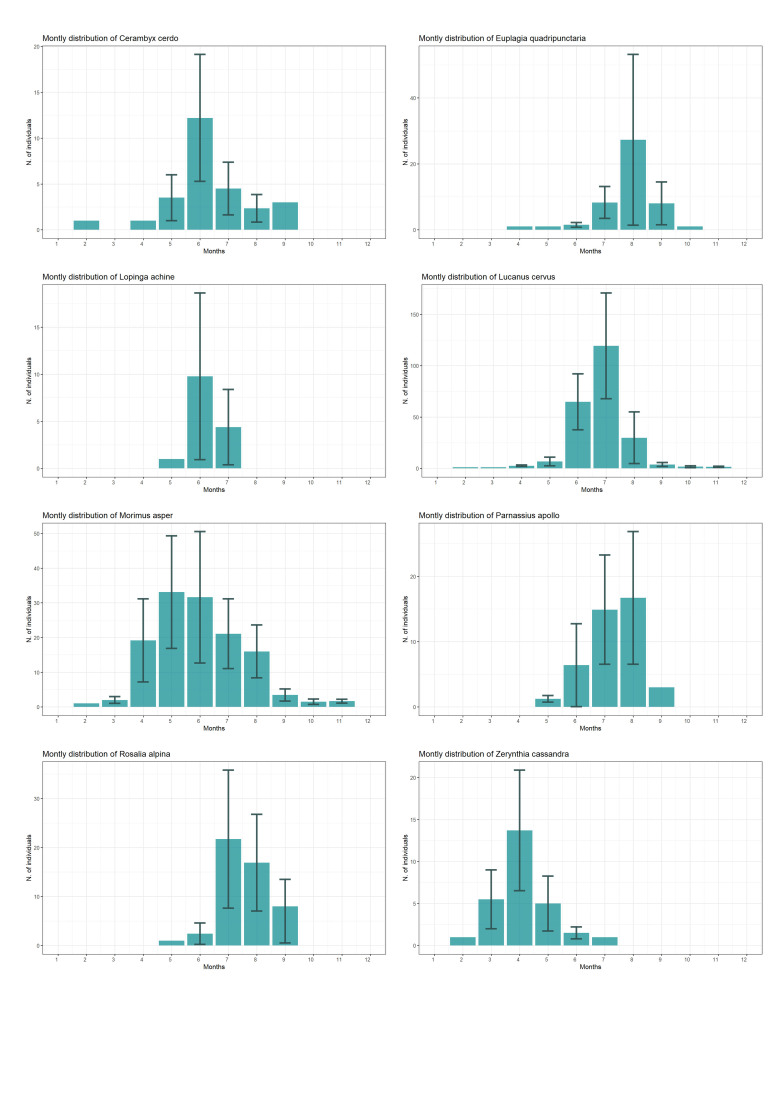
Barplot representing the mean montly distribution of the number of the recorded individuals , with the related standard error (grey bars). Only records from 2014 to 2023 were considered.

The pattern of the records collected during a year mainly reflects the months when adults of the 31 target species are usually most active, with the bulk of records having been collected in mid-July. This may be due to the fact that the majority of records are on *Lucanuscervus* (Fig. 4), which has its peak of adult activity in those weeks (Fig. 5).

**Data set 1.** 

Column label	Column description
occurrenceID	An identifier for the occurrence datum including the name of the project under which it has been collected and a unique code.
associatedMedia	URL to the repository where images related to the record are stored, freely accessible and downloadable.
catalogNumber	A unique identifier of the occurrence datum within the dataset.
basisOfRecord	The nature of the provided data.
eventDate	The complete date in which the specimen/s was/were observed.
year	The year in which the specimen/s was/were observed.
month	The month in which the specimen/s was/were observed.
day	The day in which the specimen/s was/were observed.
kingdom	The scientific name of the kingdom in which the recorded specimen/s is/are classified.
scientificName	The full scientific name, with authorship and date information if known.
order	The scientific name of the order in which the recorded specimen/s is/are classified.
family	The scientific name of the family in which the recorded specimen/s is/are classified.
scientificNameID	An identifier of the scientific name of the currently valid taxon.
verbatimIdentification	The name under which the record was uploaded by the volunteer into the project database.
genus	The scientific name of the genus in which the recorded specimen/s is/are classified.
specificEpithet	The name of the lowest or terminal specific epithet of the scientificName.
taxonRank	The lower taxonomic rank assigned to the identified specimen (e.g. subspecies, species, genus, tribe).
taxonRemarks	Comments or notes about the recorded taxon.
identifiedBy	The name of the project expert who was in charge of validating the records received from the volunteers.
decimalLatitude	The geographic latitude (in decimal degrees, EPSG:4326 - WGS84) of the geographic centre in which the specimen/s was/were observed.
decimalLongitude	The geographic latitude (in decimal degrees, EPSG:4326 - WGS84) of the geographic centre in which the specimen/s was/were observed.
geodeticDatum	The geodetic datum upon which the given geographic coordinates are based.
countryCode	The standard code for the country in which the specimen/s was/were observed.
individualCount	The number of individuals of the same species observed at the same time.
organismQuantity	The type of quantification system used for the quantity of organisms.
organismQuantityType	The type of quantification system used for the quantity of the recorded organisms.
preparations	Indication of methods used in the preparations/preservation of the samples.

## Figures and Tables

**Table 1.** List of the 31 insect taxa targeted by the MIPP/InNat initiative and included in the dataset with information about taxonomic position (genus/species, order and family) and protection status (Annex HD: Annex of the Habitats Directive in which the species is listed). Taxa originally included in the MIPP project are in bold. Remarks: *Osmodermaeremita* comprises the two subspecies *O.eremitaeremita* (Scopoli, 1763) and *O.eremitaitalicum* Sparacio, 2000, as the taxonomic position of these two taxa is still under debate (Audisio et al. 2009). The Habitats Directive lists exclusively *Morimusfunereus* (Mulsant, 1862) from the genus *Morimus*. This species is present in Italy only in a narrow part of the north-east (Carso Triestino e Goriziano within the Carnic Alps). According to several authors (Hardersen et al. 2017 and cited literature), *M.funereus* should be considered a subspecies of *M.asper* (Sulzer, 1776) and, therefore, the project collected data for both *M.a.funereus* and *M.a.asper* and the taxon is here indicated as *Morimusasper*. The two sister species *Zerynthiapolyxena* (Denis & Schiffermüller, 1775) and *Zerynthiacassandra* Geyer, 1828, were not differentiated in the InNat/MIPP initiative (i.e. reported at the genus level, as *Zerynthia* Ochsenheimer, 1816), as the two taxa cannot be separated based on wing patterns, even if they are genetically distinct (Dapporto 2010). The species *Euphydryasaurina* (Rottemburg, 1775) is treated as a single taxon, in accordance with the the recent checklist of the European Butterflies (Wiemers et al. 2018), even if Balletto et al. (Balletto et al. 2014) considered it to consist of the three different species *E.aurina*, *E.glaciegenita* and *E.provincialis* (Wiemers et al. 2018).

**Target taxon**	**Order**	**Family**	**Annex HD**
1. *Brachytrupesmegacephalus* (Lefèvre, 1827)	Orthoptera	Gryllidae	II, IV
2. ***Cerambyxcerdo* Linnaeus, 1758**	Coleoptera	Cerambycidae	II, IV
3. *Coenagrionmercuriale* (Charpentier, 1840)	Odonata	Coenagrionidae	II
4. *Coenonymphaoedippus* (Fabricius, 1787)	Lepidoptera	Nymphalidae	II, IV
5. *Cordulegastertrinacriae* Waterstone, 1976	Odonata	Cordulegastridae	II, IV
6. *Euphydryasaurinia* (Rottemburg, 1775)	Lepidoptera	Nymphalidae	II
7. *Euphydryasmaturna* (Linnaeus, 1758)	Lepidoptera	Nymphalidae	II, IV
8. *Euplagiaquadripunctaria* (Poda, 1761)	Lepidoptera	Erebidae	II
9. *Gomphusflavipes* (Charpentier, 1825)	Odonata	Gomphidae	IV
10. *Leucorrhiniapectoralis* (Charpentier, 1825)	Odonata	Libellulidae	IV
11. ***Lopingaachine* (Scopoli, 1763)**	**Lepidoptera**	**Nymphalidae**	**IV**
12. ***Lucanuscervus* (Linnaeus, 1758)**	Coleoptera	Lucanidae	II
13. *Lycaenadispar* (Haworth, 1802)	Lepidoptera	Lycaenidae	II, IV
14. *Melanargiaarge* (Sulzer, 1776)	Lepidoptera	Nymphalidae	II, IV
15. ***Morimusasper* (Sulzer, 1776)**	Coleoptera	Cerambycidae	II
16. *Ophiogomphuscecilia* (Fourcroy, 1785)	Odonata	Gomphidae	II, IV
17. ***Osmodermaeremita* (Scopoli, 1763)**	Coleoptera	Scarabaeidae	II, IV
18. ***Osmodermacristinae* Sparacio, 1994**	Coleoptera	Scarabaeidae	II, IV
19. *Oxygastracurtisii* (Dale, 1834)	Odonata	Corduliidae	II, IV
20. *Papilioalexanor* Esper, 1800	Lepidoptera	Papilionidae	IV
21. *Papiliohospiton* Géné, 1839	Lepidoptera	Papilionidae	II, IV
22. ***Parnassiusapollo* (Linnaeus, 1758)**	Lepidoptera	Papilionidae	IV
23. *Parnassiusmnemosyne* (Linnaeus, 1758)	Lepidoptera	Papilionidae	IV
24. *Phengarisarion* (Linnaeus, 1758)	Lepidoptera	Lycaenidae	IV
25. *Phengaristeleius* (Bergsträsser, 1779)	Lepidoptera	Lycaenidae	II, IV
26. *Proserpinusproserpina* (Pallas, 1772)	Lepidoptera	Sphingidae	IV
27. ***Rosalia alpina* (Linnaeus, 1758)**	Coleoptera	Cerambycidae	II, IV
28. ***Sagapedo* (Pallas, 1771)**	Orthoptera	Tettigoniidae	IV
29. *Sympecmapaedisca* (Brauer, 1877)	Odonata	Lestidae	IV
30. ***Zerynthiacassandra* Geyer, 1828**	Lepidoptera	Papilionidae	IV
31. ***Zerynthiapolyxena* (Denis & Schiffermüller, 1775)**	Lepidoptera	Papilionidae	IV
